# Understanding Barriers and Facilitators to Integrated HIV and Hypertension Care in South Africa

**DOI:** 10.21203/rs.3.rs-3885096/v1

**Published:** 2024-02-02

**Authors:** Leslie C.M. Johnson, Suha H. Khan, Mohammed K. Ali, Karla I. Galaviz, Fatima Waseem, Claudia E. Ordóñez, Mark J. Siedner, Athini Nyatela, Vincent C. Marconi, Samanta T Lalla-Edward

## Abstract

Background The burden of hypertension among people with HIV is high, particularly in low-and middle-income countries, yet gaps in hypertension screening and care in these settings persist. The objective of this study was to identify facilitators of and barriers to hypertension screening, treatment, and management among people with HIV seeking treatment in primary care clinics in Johannesburg, South Africa. Methods Using a cross-sectional study design, data were collected via interviews (n = 53) with people with HIV and hypertension and clinic managers and focus group discussions (n = 9) with clinic staff. A qualitative framework analysis approach guided by the Theoretical Domains Framework was used to identify and compare determinants of hypertension care across different stakeholder groups. Results Data from clinic staff and managers generated three themes characterizing facilitators of and barriers to the adoption and implementation of hypertension screening and treatment: 1) clinics have limited structural and operational capacity to support the implementation of integrated care models, 2) education and training on chronic care guidelines is inconsistent and often lacking across clinics, and 3) clinicians have the goal of enhancing chronic care within their clinics but first need to advocate for health system characteristics that will sustainably support integrated care. Patient data generated three themes characterizing existing facilitators of and barriers to clinic attendance and chronic disease self-management: 1) the threat of hypertension-related morbidity and mortality as a motivator for lifestyle change, 2) the emotional toll of clinic’s logistical, staff, and resource challenges, and 3) hypertension self-management as a patchwork of informational and support sources. The main barriers to hypertension screening, treatment, and management were related to environmental resources and context (i.e., lack of enabling resources and siloed flow of clinic operations) the patients’ knowledge and emotions (i.e., lack of awareness about hypertension risk, fear, and frustration). Clinical actors and patients differed in perceived need to prioritize HIV versus hypertension care. Conclusions The convergence of multi-stakeholder data regarding barriers to hypertension screening, treatment, and management highlight key areas for improvement, where tailored implementation strategies may address challenges recognized by each stakeholder group.

## Contributions to the literature

- Research has shown that there are multi-level factors that contribute to gaps in health services. Triangulating data from patients, clinic staff, and clinic leadership to assess discordances in perceived barriers to implementation is important for designing responsive implementation strategies.
- Although we identified facilitators to hypertension screening, treatment, and management that were specific to each stakeholder group, participants focused predominantly on barriers that need to be addressed to promote adoption and sustained implementation.
- Study results demonstrate how data from multiple health service stakeholder groups can be theoretically mapped to the TDF and COM-B model as the first step in intervention design.

## Background

Cardiovascular disease (CVD) is the leading cause of global mortality, accounting for an estimated 19 million deaths annually (1, 2). People with HIV (PWH) experience CVD at twice the rate of those without HIV (3, 4). The CVD burden for PWH is also disproportionately borne by low- and middle-income countries (LMICs) as more than 70% of PWH reside in LMICs, with over three-quarters of CVD deaths occurring in these settings (5–8). In African countries, CVD accounts for 37% of non-communicable disease-related deaths, and 13% of all deaths (9). Increased CVD risk for PWH is attributed to both traditional risk factors (e.g., hypertension, smoking, physical inactivity) (10–12) and HIV-specific factors (e.g., antiretroviral therapy exposure) (13, 14).

Hypertension is a leading risk factor for CVD (15), yet a majority of PWH do not receive the recommended screening and treatment (16). A large systematic review of studies on hypertension in African countries found that less than 40% of hypertensive patients were diagnosed with hypertension, less than 30% of those diagnosed received medical treatment, and fewer than 20% of those receiving treatment achieved blood pressure control (17). These gaps in the hypertension care cascade are particularly problematic in South Africa (18), which has one of the highest burden of hypertension in Africa with an estimated prevalence between 27–58% (19–21). Guidelines and resources exist for treatment and monitoring of hypertension in South Africa's public and private health sectors (22, 23), yet evidence suggests that there are gaps in care and treatment (24, 25). When compared against HIV screening and treatment metrics, hypertension screening and treatment metrics have been poor (26, 27). Given that many PWH are engaged in care, on antiretroviral therapy (ART), and virally suppressed (28), the integration of hypertension care within existing HIV care presents an opportunity to improve CVD control in this high-risk population.

To improve hypertension care for PWH in South Africa, context-specific barriers to and facilitators of hypertension care delivery should be identified. In particular, theory-based approaches to identifying determinants of care integration are needed to comprehensively understand the challenges of hypertension care integration in under-resourced HIV care settings. We report findings from a formative study aimed to identify context-specific facilitators of and barriers to hypertension care with the goal of informing the design of implementation strategies to address these.

### Methods

### Study Setting

Qualitative data collection activities were conducted across six primary care clinics in Region F of Johannesburg's Metropolitan Municipality, South Africa, a city within a province with the largest number of migrants in the country (i.e., migrants represent about 40% of the population) (29). These clinics were selected because they provide comprehensive healthcare services for PWH and are sites participating in the Integrating HIV and hEART health in South Africa (iHEART-SA) trial (UH3HL156388). iHEART-SA is a hybrid type II effectiveness-implementation trial of a multicomponent intervention to control hypertension in PWH.

### Design

We conducted a cross-sectional formative study. We used interviews and focus group discussions to gain insights into clinic staffs' knowledge, skills, and support needs to incorporate hypertension screening and treatment into routine care, as well as patients' ability to attend clinic appointments and engage in hypertension self-management.

### Ethics

Approval to conduct the study was granted by the research ethics committees at the University of the Witwatersrand (M200882) and Emory University. We used the Standards for Reporting Qualitative Research guidelines to ensure high-quality reporting (see Additional File 1) (30).

### Implementation Framework

The (TDF) was used to examine cognitive, affective, and social and environmental influences on clinician and patient behaviors related to hypertension screening, treatment, and management. The TDF was developed to assess implementation problems and has been widely used to improve understanding of health service delivery and guide quality improvement efforts (31). The framework is comprised of 14 theoretical domains that cover a spectrum of behavioral determinants, ranging from individual-level to system-level variables. The TDF can also be mapped to the COM-B model (32) across the domains of capability, opportunity, and motivation to allow for a more granular understanding of the contextual factors impacting behavior change among healthcare professionals and patients (33, 34). COM-B is the hub of the Behavior Change Wheel, a framework further comprised of corresponding intervention functions and policy categories (35). The information mapped to COM-B allows researchers to design implementation strategies to optimize the desired behavior change. This is important given that interventions targeting behavioral determinants are more likely to be effective (36).

### Participants

We purposively sampled two participants groups: clinic patients and clinics' staff. Patients were eligible if they were ≥ 18 years, spoke English or a local African language, were diagnosed with HIV and on ART, had documented hypertension, and received care at a participating clinic. Clinic staff were eligible if they worked at a participating clinic as a manager, clinician (e.g., physician, nurse, nursing assistant), or non-clinician staff member (e.g., administrator, clerk, decanting mentor, information officer) at the time of the study. To capture diverse perspectives, we recruited participants of both sexes, different ethnic groups and nationalities, ages, and professional ranks and roles.

## Data Collection Procedures

Study staff trained in qualitative research methods (AN and SL) collected data in two formats: clinic managers and patients identified based on medical records were invited to participate in in-depth interviews, while eligible clinic staff were invited to participate in focus group discussions. Interviews were conducted to understand hypertension self-management practices and attitudes towards care integration of affected individuals and how each clinic operated to support hypertension care, while group discussions served to explore clinic-level determinants of integrated hypertension-HIV care.

Semi-structured guides for each interviewee type were designed around components of the COM-B model to elicit information regarding different behaviors. For patients, interview questions focused on factors that hinder or enhance patients' ability to attend clinic and manage their chronic conditions (e.g., medication adherence, diet, physical activity). For clinic managers, interview questions assessed chronic care training opportunities within clinics, how current clinic work plans and resources create barriers to integrated hypertension-HIV care, key strategies employed to meet chronic care needs, their role in motivating the healthcare workforce, and their feedback on selected evidence-based interventions. Similarly, focus group discussion guides for clinic staff covered barriers and enablers present in accessing hypertension treatment guidelines, clinic-based factors that have hindered the implementation of hypertension screening and treatment, and strategies to enhance healthcare quality in the face of the COVID-19 pandemic. Although iHEART-SA is focused on hypertension, data collection asked questions on chronic care so that information could be used for strengthening care for other CVDs.

Due to the COVID-19 pandemic, planned in-person interviews with patients were conducted by phone in the preferred language of each participant. Data collection with clinic staff was held in-person in private outdoor spaces (in response to COVID-19 precautions). All participants provided informed consent prior to participation. Participant burden varied by the type of data collection activity, with patient interviews lasting approximately 30–45 minutes, interviews with clinic managers lasting approximately 40–60 minutes, and group discussions lasting approximately 60–90 minutes. The interviews and focus group discussions were audio-recorded, transcribed verbatim, and translated, if necessary, into English. Participant names were replaced with a unique identifier and transcripts were reviewed to ensure any identifying information (e.g., clinic name) was removed prior to data analysis. Data collection was conducted in waves, allowing the research team to review transcripts and discuss when no new codes and concepts were being generated from the data.

## Data Analysis

MAXQDA22 was used to conduct a two-stage framework analysis (37). The first stage involved a theory-driven coding process where TDF was used as the reference framework. A subset of transcripts were coded independently by two researchers to assess the fit of the data to the TDF domains. Based on the coded transcripts, agreement in coding application was assessed and in case of any discrepancies, discussions were undertaken to resolve and finalize the codebook. No major discrepancies were identified in this process and after four batches (7–10 transcripts per batch) of patient interview data and four focus group discussions per stratum of clinic staff (i.e., clinicians and non-clinicians) it was determined that code saturation had been achieved (i.e., when no new topics are identified). One additional combined focus group was conducted to ensure representation of clinics from across the region. The finalized codebook operationalizing TDF domains as they related to behaviors of patients and clinic staff was utilized to analyze the remaining transcripts.

Following this deductive analytic approach, an inductive analysis was performed to identify themes that emerged across clusters of TDF domains. Data from patients and clinic staff were analyzed separately. Data were compared across different groups of participating clinic staff and triangulated with patient-level data to determine convergence and divergence in perceived facilitators of and barriers to hypertension care in primary care settings for PWH. Patient-level data were also compared by sex and clinic site, but major differences were not identified.

## Results

In total, 46 patient interviews, seven clinic manager interviews, and nine focus group discussions (comprised of 3-7 participants each for a total of 44 engaged participants) with clinic staff were conducted across six primary care settings in Region F, Johannesburg, South Africa. Representation was sought from all six clinics; however, the senior manager was on leave in one clinic so two operational managers were interviewed instead. Saturation was reached after interviews with patients representing four clinics and after focus groups were conducted in five clinics. Key patient characteristics are summarized in Table 1. Patient interviews were conducted in Zulu (56.5%), English (10.8%), Ndebele (6.5%), Sepedi (4.4%), Setswana (4.4%), IsiXhosa (2.2%), and in a combination of these languages (15.2%).

**Table 1.** Characteristics of Patient Participants

| Characteristic | Patients ($N$=46) |
|---|---|
| | Mean (SD) |
| **Age** (Years) | 50 (8.5) |
| | $N$ (%) |
| **Sex** | |
| Female | 29 (63) |
| **Nationality** | |
| South African | 36 (78.3) |
| Zimbabwean | 9 (19.5) |
| Malawian | 1 (2.2) |

Data identified under each TDF domain were organized into themes for patients and clinic staff (see Figure 1) as they related to facilitators of and barriers to hypertension screening, treatment, and management among PWH. Both groups identified the lack of enabling resources and physical infrastructure in health clinics as the primary barrier. However, facilitators differed between the groups with clinic staff remaining that optimistic task-sharing could support care integration for PWH. Patients attributed their self-management success to the support they received from their friends and family, while being internally motivated by the fear of hypertension-related morbidity and mortality.

**Figure 1:** TDF Domains Mapped onto Qualitative Themes

**Clinic Staff Perspectives**

Interviews and focus group discussions with clinic staff generated three themes characterizing facilitators of and barriers to the adoption and implementation of hypertension screening and treatment.

***Clinics have Limited Structural and Operational Capacity to Support the Implementation of Integrated Care Models***

The limitations of existing physical and operational infrastructure to support the implementation of comprehensive care for people with chronic conditions, particularly those with hypertension, was a challenge recognized by all clinic managers and staff.

The majority of clinic staff reported that personnel shortages and a lack of medical resources impede the implementation and sustainability of hypertension screening and treatment. Clinic staff consistently emphasized that they are overburdened and have a low provider-to-patient ratio, making it difficult to consistently provide hypertension screening and treatment. A minority of individuals felt that their clinic was not conducive to performing routine blood pressure screenings because “there are extra rooms that are needed” and “extra staff [are] needed” to take vitals and provide patient counseling and support. For example, participants across several different clinics reported that the vitals room in their clinic was too small to accommodate the number of patients attending clinic on a typical day. The COVID-19 pandemic made routinely taking vitals an even greater challenge due to the lingering fear that one could contract the virus in small spaces, substantially impacting motivation. COVID-19 also impacted staff availability. One clinician shared that absenteeism due to illness placed an extra burden on the remaining staff to implement new COVID-19 screening protocols, resulting in fewer individuals available to conduct routine tasks (e.g. take vital signs). Regarding resources, blood pressure machines were frequently mentioned to be in limited supply or in disrepair.

In all represented clinics, clinic staff reported a variation in the degree of adherence to routine health screening guidelines due to staff shortages. Only representatives from one clinic reported that screenings are routinely completed for mental health, tuberculosis, diabetes, and hypertension. A few clinicians shared that screening for hypertension is often not done, or done infrequently, because HIV screening and treatment are prioritized, as best summarized by one clinic manager who stated, “Some of them get missed, we only focus on their HIV and we are missing hypertension.” A clinician from a different clinic reiterated this point, expanding to highlight the dangers inherent with these treatment gaps:

I have seen that some patients have extremely high BP [Blood Pressure] but have never received any scripts for medication for BP. I feel in the section that I am in, we focus on ART patients more than people with BP or diabetes. These patients do not get enough attention, but once the client is in front of me, we do it.

Clinic managers and staff recognized the suboptimal quality in hypertension screening and treatment, but felt the structural barriers posed substantial challenges to creating change.

In those cases when hypertension screening was performed, participants indicated that it was often dependent on the type of clinical appointment or if the patient requested to be screened. According to one staff member, in their clinic, a patient’s blood pressure is only checked if they are coming for the first time. It is understood that beyond that initial screening, a patient will only have their blood pressure taken if they are making an appointment regarding hypertension care needs, as expressed by one clinician who stated:

The next time you come back, you have no blood pressure [taken], you have come back for something else which is different, and your blood pressure does not get done…The previous time you did not have blood pressure, what you are complaining about today has nothing to do with blood pressure. Then why am I still going to check your blood pressure when I've got people whose blood pressure needs to be checked because they're here for that?

Other operational challenges shared across clinics included patients skipping the vitals room to avoid waiting in long lines and the challenges related to continuity of care and data management when the filing system is disorganized. A minority of participants perceived that their clinics were already equipped to implement hypertension screening and treatment.

#### *Education and Training on Chronic Care Guidelines is Inconsistent and often Lacking across Clinics*

Nearly all clinic staff identified a lack of training and knowledge of evidence-based chronic care guidelines as a reason why routine hypertension screening and treatment was not being implemented in their clinical settings. Clinicians reported having access to the South African Department of Health guidelines and essential drug lists within the clinic or on their phones, but that the onus of responsibility falls on the individual to note where changes have occurred. Another barrier to screening was the belief that PWH do not need to be prioritized for cardiovascular risk assessment. Clinic staff also highlighted that knowledge of routine hypertension screening and treatment is necessary to support clinicians, or to adopt task-shifting models when there are staff shortages. This was complimentary to the perspective of most clinicians, who expressed that staff members and community health workers need training opportunities that will empower them to support routine hypertension screening.

Clinic staff reported that their knowledge base for chronic care among PWH was derived through a combination of formal educational programs, periodic training in-clinic, and through information shared by health promoters.  Participants agreed that each clinic was staffed with individuals with varying levels of knowledge and skills related to chronic disease care. In reflecting on clinical training needs, clinic managers voiced that there was a need for more training on chronic care guidelines, particularly for nurses who are non-NIMART (Nurse Initiated Management of Antiretroviral Therapy) trained, “Because we also get some people that are not NIMART trained that are assisting with the chronic patients.” And while clinic staff shared that foundational information sessions were facilitated, for example, by the district health management on the chronic care guidelines set forth by South Africa’s Department of Health, they also noted that chronic care training was not hypertension focused and that there was no stated expectation to screen each patient for hypertension. In-service training can also target different audiences, so that staff only attend if it is relevant to their job responsibilities. Despite the recognized need for and perceived value of in-clinic training sessions on chronic disease care, half of participants reported that training was difficult to attend because the sessions are time consuming and infrequently offered.

The variation in training within clinical settings creates a system whereby clinicians become reliant on their peers who have more training in select clinical competency areas. For example, according to one nurse, “when I encounter an issue [hypertension], I may have to take that patient to a primary health care (PHC) trained sister to deal with the patient, and because of this, we are not able to provide a one-stop-shop kind of care. So, our training is limited.” Non-clinician staff members, on the other hand, report that they are unable to operate outside of the role they were trained for, such as data capturers and analysts, but are often asked to do tasks beyond their skill level to support the implementation of quality care practices.

#### *Clinicians have the Goal of Enhancing Chronic Care within their Clinics but First Need to Advocate for Health System Changes that will Sustainably Support Integrated Care*

The integration of hypertension screening and care for PWH was largely supported by clinicians, though some exhibited a reluctance due to beliefs around anticipated implementation challenges related to material and human resources. Anticipated barriers included imposing additional responsibilities on the existing staff (especially for data capturers who would need to collect and integrate new data elements into the metrics they currently report on for the clinics), the time-consuming nature of such a model, possible resistance from non-clinician staff members, and uncertainty around the ability to adhere to the guidelines given the high volume of patients at each clinic. Clinicians reported conflicting information on who was responsible for taking vitals, with nearly everyone agreeing that staff were not able to consistently measure blood pressure due to high patient volume. Individuals who had a positive outlook on integrated care, however, believed that this care model has the potential to reduce staff workload and shorten patient waiting times. Clinic administrators felt this care model would work because tasks could be divided amongst the staff, thus not overburdening any one individual. Clinical managers expressed receptiveness to integrated hypertension-HIV care, however, voiced that it should be introduced and operationalized by the Department of Health. Only representatives of one clinic shared that they are already working towards the integration of hypertension and HIV services.

All clinics' staff were aware of the standard workflow in their clinical settings and bottlenecks in clinic operations that required improvement for integrated hypertension-HIV care to successfully operate (e.g., creation of a shared space for vitals checkup, integrated electronic filing system in place across clinics). Clinic staff shared how they were actively working to overcome these implementation barriers. For instance, in planning for this integration, some clinics were actively advocating for equipment, having clinicians conduct rotations with a chronic care focus, and extending chronic care-focused training to the managers. Participants from a few clinics also indicated receptiveness towards the idea of having technological innovation adopted to promote an integrated care approach, for example, an electronic system to capture and report patient data, a digital treatment guideline for ease of access and use, and an automated reminder-based system for patients to reduce workload and burden on the staff. Individuals from two clinics were aiming to have all patients pass through their vitals room and to provide blood pressure machines to the patients to ensure proper monitoring outside of the clinic setting. None of the clinics had systems in place to remind clinicians to complete tasks for hypertension screening and care.

To reinforce the training on hypertension care, clinic staff agreed that incentive structures and strategies to motivate staff were needed. Some clinics had a recognition system to improve adherence to care guidelines, but individuals from one of those clinics reported that it was not effective and that they would prefer monetary incentives instead. Additionally, clinicians reported that the typical top-down approach to implementing changes in the clinical environment was demotivating. They expressed that being a part of decision-making processes and having opportunities to provide feedback to senior administrators and managers is an important aspect of work culture.

#### Patient-level IDI Data

Interview data with PWH and hypertension generated three distinct themes characterizing existing facilitators of and barriers to clinic attendance and chronic disease self-management.

##### *The Threat of Hypertension-related Morbidity and Mortality as a Motivator for Lifestyle Change*

Most of the patients reported that their motivation to modify their behavior towards hypertension management originated from their fear of hypertension-related adverse health outcomes. Any effort to regulate their behavior, set goals, or adopt preventive practices was therefore primarily to avoid the potential life-threatening consequences of hypertension. For example, one patient shared that if they did not take their medication every day, they feared that they would have a stroke, while another shared that they would end up hospitalized. Several patients shared accounts of knowing someone who died as a result of uncontrolled hypertension and the desire to avoid the same fate. One patient in her early 40’s reflected on what motivates her to maintain a healthy lifestyle, sharing:

What encourages me….is that I have seen so many people suffer the consequences of BP without knowing that they even had it, people find out when they already have a stroke. One of my colleagues fell and passed out last week and we called an ambulance for her, they told her the BP was too high.

Participants also reported wanting to stay healthy to avoid having to go to the clinic. As one patient explained, “They monitor your weight too and ask when it has changed drastically. When my BP is not controlled, the punishment is always that I need to be back in a month…it keeps me motivated and accountable.” The sentiment that clinic visits were burdensome and stressful was shared widely. A minority of patients felt less optimistic about managing their condition, even with help from their clinicians, as they believed that their hypertension “will not be cured.”

Patients described a range of goals they believed would help them control their blood pressure, including adopting strategies that focused on reducing unhealthy practices (e.g., skipping meals, feeling angry) and enhancing healthy ones (e.g., attending clinic appointments). Adherence to medication was the most common form of behavior regulation reported among the participants, followed by dietary change (e.g., reducing salt intake, increased vegetable intake), staying hydrated, which they believed helped control anger and stress, and increasing physical activity. A number of individuals described having to develop new routines to build physical activity into their schedule. For instance, one patient reported substituting short taxi rides with walking. A minority of participants reported that they did not regulate their diet and solely concentrated on taking their medications as prescribed.

Even when individuals recognized that they were not meeting their hypertension self-management goals, they voiced an intention to make positive lifestyle changes. Most participants expressed their desire to live a long and healthy life so that they could care for their children and grandchildren and were confident they could make incremental progress to achieve their health goals.

##### *The Emotional Toll of Clinics’ Logistical, Staff, and Resource Challenges*

In describing their typical experiences seeking hypertension treatment at their respective clinics, nearly all patients described clinics as unorganized (e.g., lost files, inconsistent blood pressure screenings) and inadequate in terms of staffing and resources (e.g., broken blood pressure machines). Many patients felt long wait times were a barrier to seeking care, especially during COVID when lines were moved outside, as people felt there was no privacy or shelter from the elements. Participants recommended having larger waiting areas indoors, separate lines for chronic care appointments, and community-based collection points for medication pick-up to combat these issues. Patients reported mixed satisfaction with clinics based on encounters with staff, with some advocating for clinicians to be trained in communication skills to improve the quality of care. Factors that motivated patients to come to clinic included already being familiar with staff members, proximity to their home, and having their medical history already on file at the clinic.

A subset of patients reported positive experiences with their clinic that motivated them to return. For example, some individuals were able to get prescriptions filled for longer periods than the standard one-month supply which allowed for less frequent visits. Others felt encouraged by the feedback from their clinicians and felt cared for by the clinic staff, as one patient in her 60's explained:

It is that I receive my treatment the way I am supposed to receive it and even if I missed my date, they call me and ask why I did not come. That shows that they care about us and that will always make me continue seeking care here.

The emotions associated with attending clinic were compounded by individuals' emotional experiences with their chronic conditions. One person described the diagnosis of hypertension as a traumatic experience, while many others felt that the period following the diagnosis to be the most frustrating. Individuals reported having their clinician's attitude towards them worsen as a result of their hypertension diagnosis, experiencing extended wait times, and discontent with the record-keeping system.

#### *Hypertension Self-management as a Patchwork of Informational and Support Sources*

Hypertension self-management practices were described as being facilitated by a range of informational and supportive resources, but patients highlighted the need for more clinic-based counseling and education on hypertension. When asked whether someone else has an impact on their hypertension management, few patients reported that they are able to manage it independently. Others relied on family members to collect and remind them to take medications and to provide dietary support, as one patient in his 40's shared, "They always make sure that when it is that time, they ask me if I have taken my medication and if not, they remind me to take them there and then." Patients also received informational and emotional support from community-based patient support groups. Across all participants, people reported relying on limited channels of information for hypertension self-management, which included, their clinician, individuals on the same medication, traditional healers, and information found through online research. For most patients, the information that was provided by clinicians was focused on dietary requirements, medication adherence, and consequences of uncontrolled hypertension. All patients expressed an interest in receiving and learning how to operate a blood pressure machine in order to track their blood pressure at home.

#### Data Triangulation across Participant Groups

Factors enabling hypertension screening, treatment, and patient self-management were unique to each stakeholder group based on the target behavior. However, consistency was found in the factors that may hinder adoption and implementation of hypertension screening and treatment among PWH, as evidenced by the convergence in findings from both clinic staff and patients (see Table 2). Both groups acknowledged the lack of infrastructure and clinical operations supporting routine hypertension screening as barriers to the implementation of an integrated care approach. An area of discordance was identified in terms of disease priority. Patients appeared to consider hypertension more severe compared to HIV, while clinic staff considered HIV to be the primary concern among patients with both diseases.

**Table 2.** Summary of Patient and Clinic Staff Reported Barriers to Hypertension Screening, Treatment, and Management

| COM-B Component | TDF Domain | Barriers from the Perspective of Clinic Staff | Barriers from the Perspectives of Patients |
|---|---|---|---|
| Capability | Knowledge | • Lack of guideline knowledge | • Lack of health education on hypertension and role of medication in BP control |
| Capability | Skills | • Lack of in-service training | • Lack of skills to operate a home BP machine |
| Capability | Behavioral regulation | • No supporting data | • Advanced age, conflicting schedules, and limited finances to achieve an improved diet, physical activity levels, medication adherence, and clinic attendance. |
| Capability | Memory, attention and decision processes | • Competing clinical priorities | • Forgetfulness regarding medication adherence |
| Opportunity | Environmental resources and context | • Inadequate space for taking vitals<br>• Lack of functional blood pressure machines<br>• Lack of human resources<br>• Limited time during patient encounters<br>• Lack of data management for quality improvement | • Distance to clinic<br>• Long clinic wait times<br>• Disorganized file system |
| Opportunity | Social Influences | • No expectation to take BP for each patient<br>• Being asked to take on new roles outside of job responsibility to support integrated care | • Mistreatment from family and friends due to diagnosis or lifestyle changes resulting from hypertension self-management<br>• Limited sources for health information |
| Motivation | Emotions | • Apprehension towards integrating hypertension care given material and human resource limitations | • Fear of hypertension-related morbidity and mortality<br>• Negative beliefs about what their in-clinic experience will be |
| Motivation | Goals | • Awareness of chronic care needs for PWH, with mixed views regarding prioritization of hypertension care | • No barriers identified |
| Motivation | Intentions | • Identification of resource needs and action planning to meet patients' chronic care needs | • No barriers identified |
| Motivation | Reinforcement | • No reminders to complete hypertension screening and care<br>• Ineffective incentivization methods | • No barriers identified |
| Motivation | Beliefs about consequences | • HTN not seen as an issue among PWH | • No barriers identified |
| Motivation | Optimism | • Mixed confidence in ability to implement hypertension care guidelines | • Pessimism related to belief about hypertension consequences |
| Motivation | Beliefs about capabilities | • Pressure to work quickly results in inability to take each patient's blood pressure | • Access to a BP machine to self-monitor at home |
| Motivation | Professional role and identity | • Taking vitals is considered a task someone else could complete | • No supporting data |

## Discussion

The study used mixed qualitative methods to understand the facilitators of and barriers to hypertension screening, treatment, and management among PWH seeking treatment in primary care clinics in Region F of the city of Johannesburg, South Africa. Using a two-step analysis guided by the TDF, perceived behavioral determinants were compared among different groups of participating clinic staff and triangulated with patient-level data to determine convergence and divergence in perceptions between these two groups.

## Clinic-level Enablers and Barriers

The primary barriers to implementing hypertension screening and treatment identified in this study were the lack of operational capacity and siloed flow of operations in clinics and the limited value placed on hypertension care guidelines. Nearly all clinic actors expressed that clinic infrastructure and operational procedures were not currently suitable for implementing HIV and hypertension chronic care. These findings align with qualitative studies conducted in African countries, including South Africa, that assessed barriers to integrated hypertension-HIV management in clinics and found that non-functioning blood pressure machines, inadequate planning for care integration, and lack of anti-hypertensive medicines hindered implementation success (38–40). Clinicians in this study also reported that routine blood tests and blood pressure checkups were not implemented with fidelity. Neglecting to screen PWH for hypertension puts them at risk of developing serious hypertension-related complications (e.g., severe headache, stroke) (41), otherwise preventable conditions. To address these challenges and ensure quality care for both HIV and comorbid chronic conditions (e.g., hypertension), context-specific implementation strategies are necessary to integrate HIV and chronic care in South Africa.

This study identified additional barriers to implementation that varied based on provider characteristics and training opportunities afforded to them within their clinical context. Inadequate training on hypertension care guidelines was frequently cited as a barrier in hypertension care management. The importance of providing disease-focused education and training opportunities is underscored by findings from Musinguizi and colleagues who found that healthcare providers without specialized training and skills in chronic care are forced to attend to patients with chronic illnesses due to staff shortages, resulting in suboptimal clinic performance in terms of care management (42). Establishing a monitoring and evaluation system (e.g., audit and feedback) that keeps clinicians' adherent to hypertension screening and treatment guidelines is one approach that has been proposed to help combat clinical inertia (39).

The ability to practice task-sharing to some degree was identified as a key factor in facilitating the adoption and implementation of hypertension screening and care in primary care clinics. Clinicians in our study reported instances where tasks were referred to other professionals based on the level of complexity and specialization required in accordance with clinic protocols. This approach was found to be beneficial in streamlining patient workflows and allowing for successful BP screening and hypertension care with limited staff. Studies undertaken in LMICs have tested the effectiveness and feasibility of task-sharing interventions for integrating the management of noncommunicable diseases with this approach and have shown success in improving care management and health outcomes (e.g. increased uptake of medications and reductions in blood pressure (43)). Further research are needed to fully understand the effects of task-sharing on healthcare professional burnout and patient-centered outcomes.

## Patient-level Enablers and Barriers

Previous studies from South Africa have demonstrated a lack of knowledge among patients about hypertension, which contributed to poor disease self-management (44, 45). In this study, patients reported that the threat of hypertension-related morbidity and mortality acted as a driving force for them to adopt new health behaviors. There are a limited number of studies exploring patient motivation to practice hypertension self-management within the African cultural context; however, those studies that do have demonstrated that patients are motivated by knowledge of how uncontrolled hypertension will impact their health. A study conducted in a primary care hospital in Nigeria among hypertension patients found that increased awareness of the consequences of uncontrolled hypertension facilitated self-management practices for the condition (46). Other international studies provide evidence that modifying health behaviors is often driven by the fear of poor health outcomes (47, 48). These results suggest that efforts to educate people about the importance of lifestyle changes and the potential consequences of neglecting proper hypertension care management should be prioritized.

## Strengths and Limitations

The application of the TDF in this study afforded an opportunity to examine patient- and clinic-level determinants of hypertension screening, treatment, and management for PWH. The involvement of multiple stakeholders - patients, clinic managers, clinicians, and non-clinician staff - provides a comprehensive understanding of implementation challenges that can be anticipated and enables the triangulation of data, thereby increasing the reliability of the findings. This study is limited by the fact that interview and discussion guides were not developed to elicit data on each TDF domain, which likely accounts for the absence of data for several TDF domains. The study was conducted in an urban setting characterized by a large migrant population, limiting the transferability of study findings. Additionally, the COVID-19 pandemic caused interruptions and changes in data collection methods that may have hindered participation and altered participant's perceptions regarding health services.

### Conclusion

Integrated care models continue to gain increasing global attention for their potential benefits to improve chronic disease control, patient satisfaction, and service delivery. This study details how multi-level barriers may hinder the successful integration of hypertension care into routine HIV care and will inform the design of implementation strategies aimed to promote the adoption and implementation of guideline-recommended hypertension screening and management practices in HIV care.

### Abbreviations

CVD—Cardiovascular disease

HIV—Human immunodeficiency virus

PWH—People with HIV

LMIC—Low- and middle-income country

TDF—Theoretical Domains Framework

COM-B—Capability, Opportunity, Motivation, Behavior

ART—Antiretroviral therapy

## Declarations

#### Ethics approval and consent to participate

Ethical committee approval was obtained in the United States from Emory University and in South Africa from the University of the Witwatersrand. Participants provided informed consent prior to participation.

#### Consent for publication

Consent form allowed the use of anonymized quotations in publications.

#### Availability of data and materials

The datasets generated and analysed during the current study are not publicly available due confidentiality policies but are available from the corresponding author on reasonable request.

#### Competing Interests

VCM has received investigator-initiated research grants (to the institution) and consultation fees from Eli Lilly, Bayer, Gilead Sciences, Merck, and ViiV. The remaining authors declare that they have no competing interests.

#### Funding

The study received support from the NIH/NHLBI (UG3HL156388). VCM has received funding support from NIH/NHLBI (UG3HL156388) and the Emory Center for AIDS Research (P30AI050409) for work related to this manuscript. The funder had no role in designing, conducting, or interpreting study findings.

#### Author's Contributions

LJ led the conception and design of the study and was closely involved in data analysis, interpretation, and writing of the manuscript. SK led data analysis and interpretation and was involved with FW in writing the manuscript. AN and SL were involved with data collection. KG, CO, MA, VM, AN, MS, and SL were involved with the design of the study, participated in data interpretation, and revised the manuscript critically for intellectual content. All authors read and approved the final manuscript.

### Acknowledgements

The iHEART-SA study team would like to thank all study participants.

## Figures

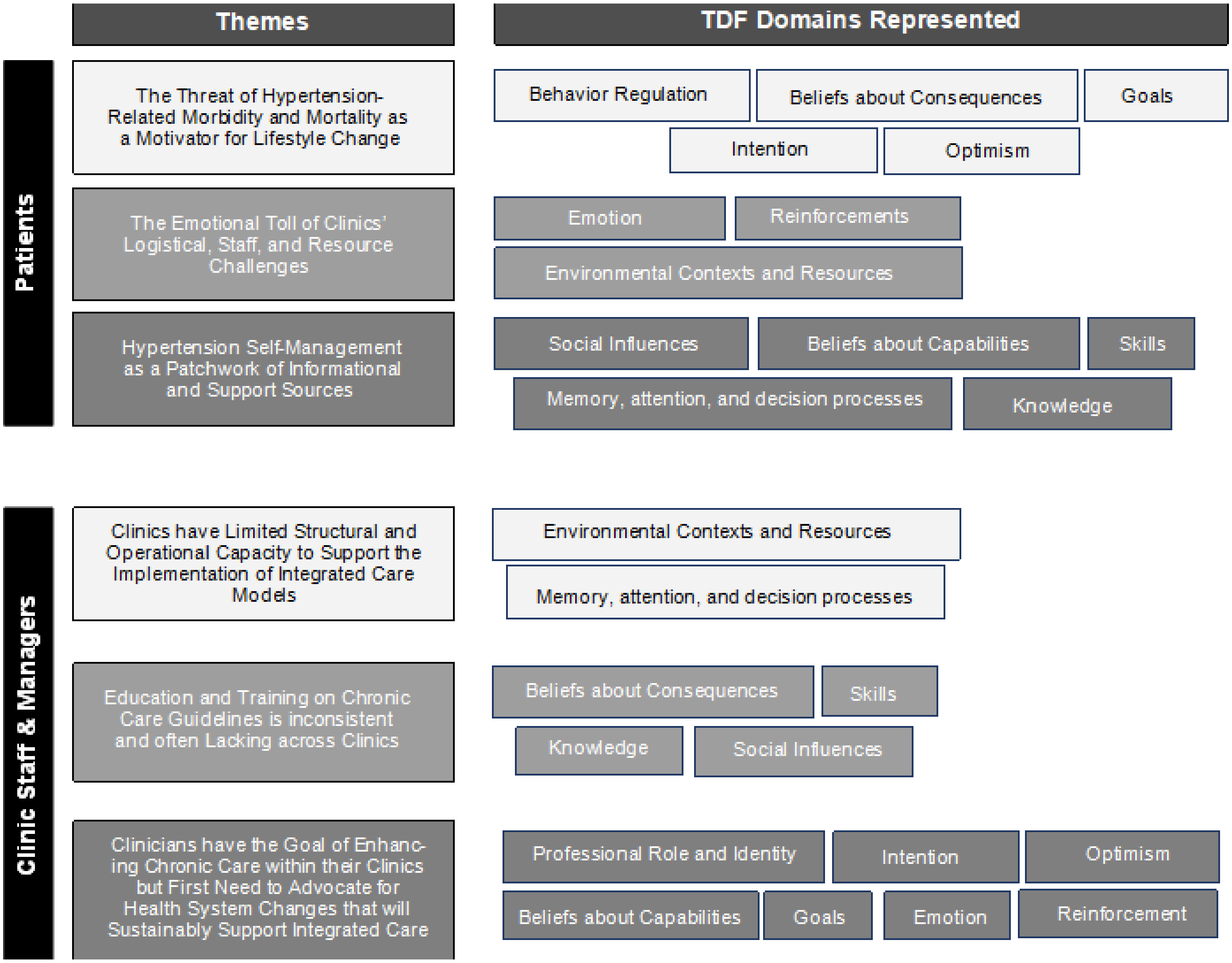

**Figure 1**

TDF Domains Mapped onto Qualitative Themes

*Note:* TDF constructs 'behavioral regulation' and 'professional role and identity' were not identified in clinic staff and patient data, respectively.

## Supplementary Files

This is a list of supplementary files associated with this preprint. Click to download.

- SRQRChecklist.docx
